# Small access postaural parotidectomy: an analysis of techniques, feasibility and safety

**DOI:** 10.1007/s00405-015-3691-9

**Published:** 2015-06-30

**Authors:** Anthony Po-Wing Yuen

**Affiliations:** Hong Kong Head and Neck Cancer Surgery Centre, Room 1213, Central Building, 1 Pedder Street, Central, Hong Kong, SAR China

**Keywords:** Parotidectomy, Parotid tumor, Minimally invasive parotidectomy, Postaural parotidectomy

## Abstract

For cosmetic consideration of parotidectomy, the surgical approaches have evolved from Blair incision through modified facelift incision to postaural-hairline incision. The present study aims at evaluating the feasibility and safety of the new technique of postaural approach. Parotidectomy was performed with a 4–5 cm incision in the postaural sulcus. There were 69 patients who were assessed pre-operatively feasible for consideration of the postaural parotidectomy. There were 56 (81 %) patients who could have the postaural parotidectomy successfully without complications. The minimally invasive postaural approach is a further step in cosmetic consideration of parotidectomy. It is a feasible and safe approach for most small to medium size benign parotid tumors located in the mid and lower pole regions of the parotid gland.

## Introduction

Parotidectomy has been performed commonly using the Blair approach with a large S shape incision from the lateral face over the parotid gland down to the upper neck. Although this incision may heal with quite unnoticeable scar in patients with white skin color, this traditional incision has a poor cosmetic result of a long visible permanent scar on the face and neck particularly in patients with yellow, brown or black color skin. The long visible scar can lead to long-term psychosocial complications of the patients. Ciuman et al. have shown that cosmetic discontent of surgical scar and deformity after parotidectomy significantly affected adversely symptom-specific and general quality of life scores [1]. Patients may refuse operation because of the unacceptable facial scar, not until the tumors have grown into large size or develop clinical signs of malignancy.

With due consideration of the cosmetic problem of traditional Blair approach, a more cosmetically acceptable modified facelift approach has been increasingly performed since its publication about 30 years ago [2–7]. The modified facelift approach has incisions in three regions including preaural, postaural and hairline. Of the incisions in the three regions with this facelift approach, the postaural incision is almost invisible as it is hidden by the auricle. Facelift approach still has visible scar in the preaural and hairline regions. The hairline scar tends to become hypertrophic and visible particularly in male patients with short hair. Wasson et al., Bianchi et al. have shown that modified facelift incision had better cosmetic outcome compared with Blair incision [8, 9].

The author has been trained in performing parotidectomy using the traditional Blair incision approach and then started using modified facelift approach since 1997. With experiences gained in the modified facelift approach, the author started to use postaural-hairline approach in 2005. The postaural-hairline approach can avoid the preauricular scar of modified facelift approach. The postaural-hairline surgical techniques and results were published in 2010 [10]. The postaural-hairline approach is cosmetically better than the modified facelift approach and is applicable in about 80 % parotidectomy [10].

With experiences gained in postaural-hairline approach, the author started using an even smaller incision for parotidectomy with the postaural incision alone since 2011. The present study aims at evaluation of the feasibility and safety of this small access postaural approach.

## Materials and methods

All patients presented with parotid mass were evaluated with ultrasound and ultrasound guided fine needle aspiration cytology. Patients were informed that definitive diagnosis could not be achieved accurately with clinical features and cytology for parotid mass; surgery was necessary for persistent or enlarging mass for both definitive histological diagnosis and treatment. Patients were advised that partial parotidectomy would be performed and the surgical specimen would be sent to pathologist for intraoperative frozen sections. Frozen section might not be able to confirm the definitive pathology; however, it could indicate accurately whether the lesion was benign or malignant in nature. If the frozen section pathology was suggestive of benign in nature, partial parotidectomy was adequate. If the frozen section pathology was suggestive of malignant tumor, total parotidectomy would be performed immediately after frozen section. Of all patients who were considered potentially feasible by the author for small access postaural parotidectomy, they were given the options of Blair incision, facelift incision, postaural incision with extension incisions and small access postaural incision. All patients in this study opted for attempt of small access postaural parotidectomy with consent to proceed for extension incisions, flap reconstruction and nerve graft to be decided necessary intraoperatively.

**Fig. 1:**
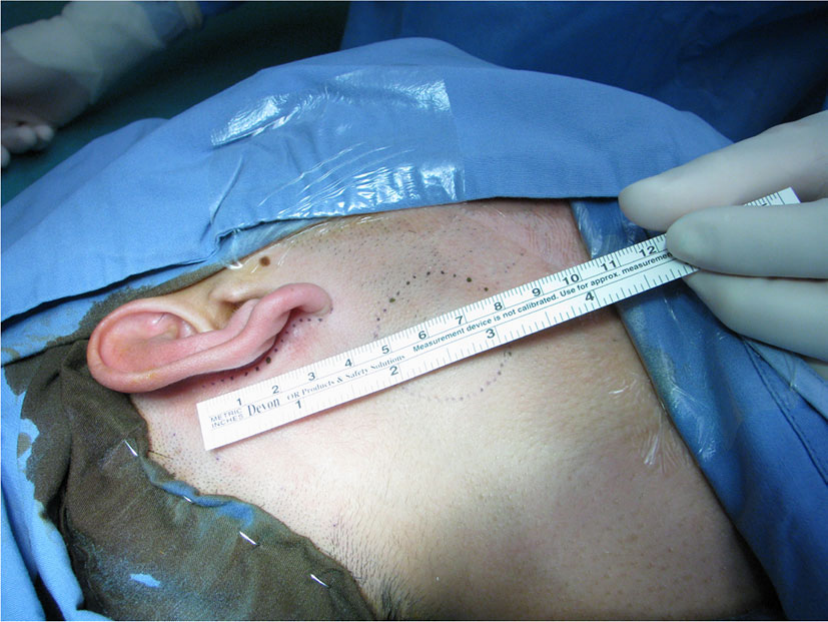
A 4 cm postaural incision for parotidectomy of a 3 cm pleomorphic adenoma (*circular dots*) of right parotid

**Fig. 2:**
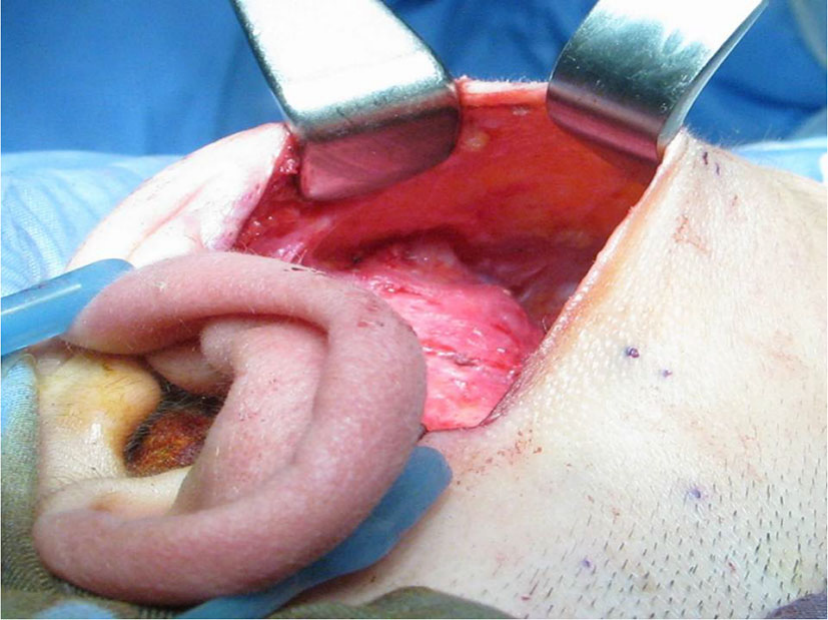
The skin is undermined to expose the sternomastoid muscle and parotid gland

**Fig. 3:**
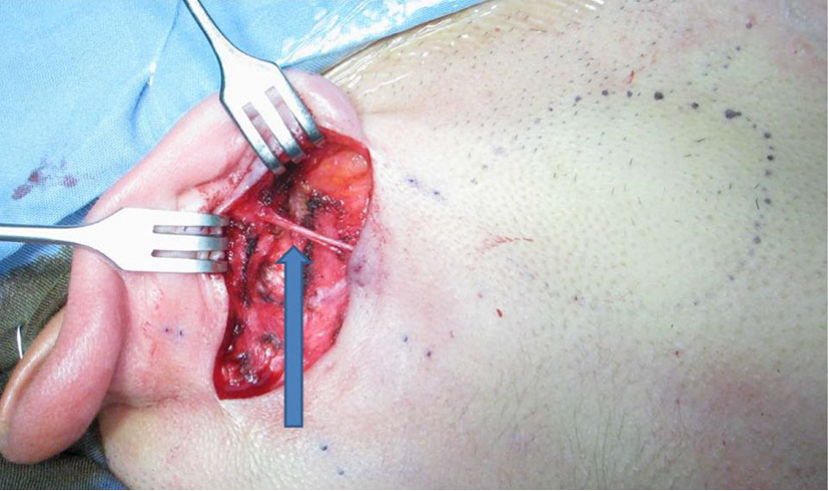
The auricular branch of the greater auricular nerve (*arrow*) is preserved and dissected free from the parotid gland

**Fig. 4:**
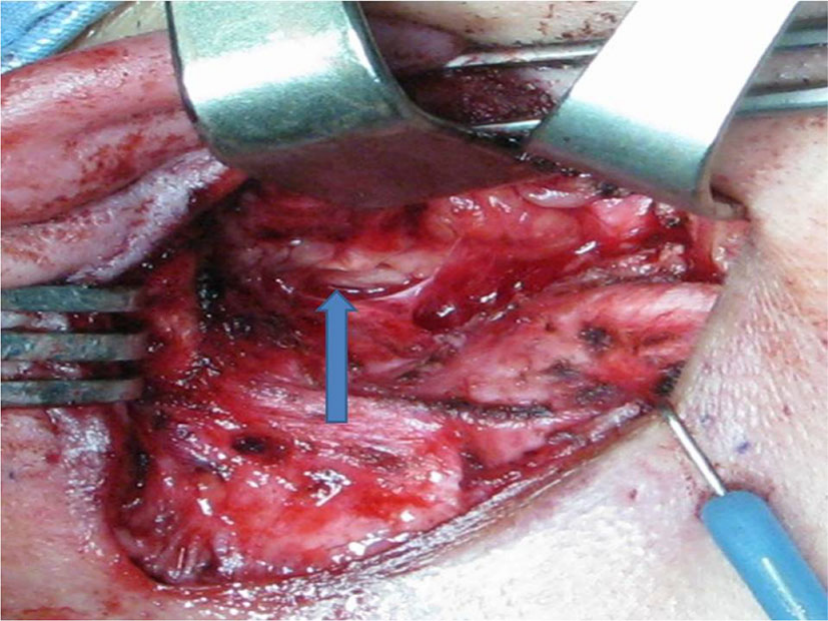
The facial nerve trunk (*arrow*) is identified at its exit point from the stylomastoid foramen at the tympanomastoid fissure

**Fig. 5:**
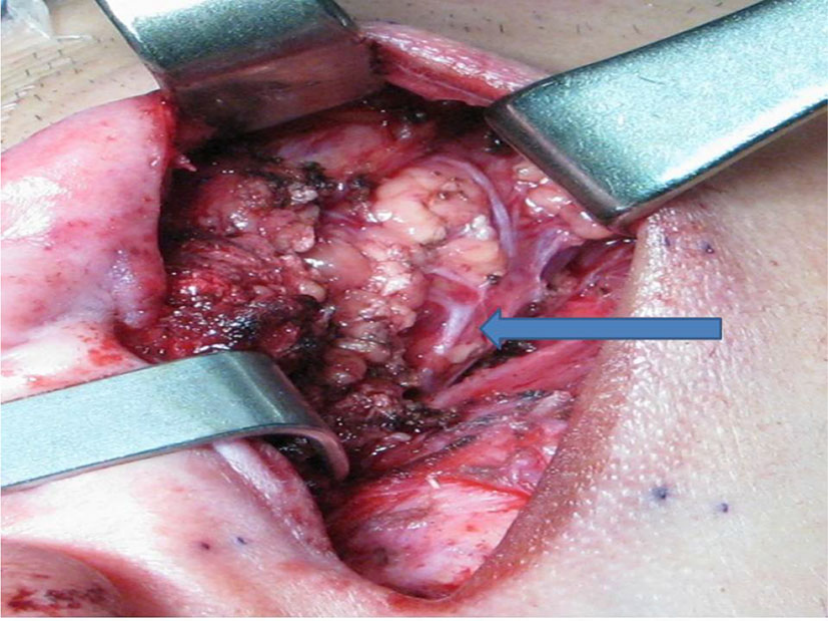
Partial parotidectomy is completed, normal parotid tissue and facial nerve (*arrow*) are preserved

Small access parotidectomy has an incision 4–5 cm long in the postaural sulcus as shown in Fig. 1. The skin is undermined to expose the sternomastoid muscle and parotid gland as shown in Fig. 2. The subsequent steps of parotidectomy are performed using the same radiofrequency bipolar cutting and coagulation techniques as in postaural-hairline approach previously published in 2010 [8]. The auricular branch of the greater auricular nerve is preserved and dissected free from the parotid gland as shown in Fig. 3. The parotid gland is mobilized free from the sternomastoid muscle, posterior belly of digastric muscle, tragal cartilage and tympanomastoid fissure. The facial nerve trunk is identified at its exit from the stylomastoid foramen in the tympanomastoid fissure as shown in Fig. 4. The parotid gland is dissected along its facial nerve branches to remove the parotid tumor. Partial parotidectomy with 2 mm resection margin is performed for benign tumor as shown in Fig. 5. For malignant tumor, total parotidectomy is performed. The extended sternomastoid flap is utilized if necessary to fill-up the surgical defect as described in my previous publication [8]. The postaural approach is attempted in all feasible patients. In case the exposure is found inadequate intraoperatively, extension incisions can be performed with either the preaural extension and/or hairline extension as decided appropriate to improve the exposure for further dissection.

The first small access postaural approach was performed in March 2011. From March 2011 to November 2013, the author has performed a total of 79 parotidectomies. In this period, 10 (12 %) patients were excluded from this surgical approach in pre-operative assessment for various reasons: one patient had recurrent malignant parotid tumor with prior Blair incision parotidectomy, the old scar was used; an elderly patient with dementia and a 7 cm deep lobe pleomorphic adenoma wished a quick operation without consideration of scar problem, and the Blair incision was used; 5 patients had malignant parotid tumor in which radical parotidectomy (2 also needed facial nerve graft) was performed with modified facelift incision; one patient had deep lobe parotid haemangioma in which feeding vessels were ligated without parotidectomy; one patient had a 8 cm deep lobe tumor which was considered too large for postaural approach and a modified facelift approach was decided pre-operatively; one patient had accessory parotid pleomorphic adenoma overlying the parotid duct in which a transoral approach was performed. After exclusion of these 10 patients, there were 69 patients included in this study, and they were considered pre-operatively for possible attempt to perform minimally invasive postaural approach with reservation of hairline and/or preaural extensions if necessary.

## Results

Of the 69 patients recruited for postaural small access approach, 56 (81 %) patients had successful parotidectomy using the small access postaural incision alone without extension. Of the 13 patients who needed extension incision, there were 7 preaural extension, 2 hairline extension, 4 both preaural and hairline extension. The details of these 13 patients are shown in Table 1. Of these 13 patients who required various extension incisions as decided necessary intraoperatively, there were 9 superficial and 4 deep lobe lesions. The reasons for extension incisions were difficult dissection through the small postaural wound in 6 patients (3 due to deep lobe location, 2 due to large tumor size of 6 cm, 1 due to facial nerve Schwannoma), inadequate exposure in 6 patients (3 too far anterior location, 3 too far upper pole location), and combination of difficulty of dissection in deep lobe and inadequate exposure in too far anterior location in 1 patient.

**Table 1:** Details of 13 patients who needed extension incisions

Extension	Pathology	Location	Size (cm)	Reason for extension incisions
Preaural	PA	Deep lobe	5	DD deep lobe
	PA	Deep lobe, partially embedded in masseter muscle	3	DD deep lobe and IE anterior
	Spindle cell tumor	Lower pole to upper pole	6	DD too large, IE upper
	Lymphoepithelial lesion	Parotid duct	1.5	IE anterior
	PA	Upper pole	2	IE upper
	Lipoma	Mid to upper pole	5	IE upper
	PA	Upper pole	5	IE upper
Hairline	Warthin	Lower pole	6 and 2	DD too large
	PA	Anterior	2	IE anterior
Preaural and hairline	PA	Deep lobe	5	DD deep lobe
	PA	Deep lobe	4	DD deep lobe
	PA	Anterior	2	IE anterior
	Facial nerve schwannoma	Centre	4	DD

*PA* pleomorphic adenoma, *DD* difficult dissection, *IE* inadequate exposure

Of the 56 (81 %) patients who had successful complete removal of the parotid mass with the minimally invasive postaural approach. There were 27 male and 29 female patients. The mean age was 40 years (range 27–67 years). There were 37 pleomorphic adenoma, 6 basal cell adenoma, 5 Warthin tumor, 1 oncocytoma, 3 lymphoid hyperplasia, 1 lymphoma, 1 low grade mucoepidermoid carcinoma, 1 lipoma and 1 Kimura disease. Intraoperative frozen sections were all correct in differentiating benign and malignant nature of the parotid mass, and therefore the correct parotidectomy procedures were performed as appropriate. The mean tumor size was 3 cm (range 1–6 cm). There were 46 superficial lobe and 10 deep lobe tumors.

Twenty (36 %) patients needed extended sternomastoid flap for reconstruction of the surgical defect. There was no facial palsy (temporary or permanent), wound infection, Frey’s syndrome or tumor recurrence in all 69 patients. All patients were satisfied with the small access postaural wound with cosmetic result achieved to their expectation, and no patient had any complaint about the cosmetic problem of the scar.

With reference to the total of 79 parotid surgeries in this period of study, there were 56 (71 %) small postaural incision, 11 (14 %) modified facelift incision or postaural incision with both preaural and hairline extensions, 7 (9 %) postaural incision with preaural extension, 2 (3 %) postaural incision with hairline extension, 2 (3 %) Blair incision and 1 (1 %) transoral incision.

## Discussion

The results of the present study show that small access parotidectomy with a 4–5 cm long postaural incision alone without preaural and hairline extension is feasible and safe in most patients with small to medium size (mean 3 cm and up to 6 cm) benign parotid tumor of both superficial and deep lobe. This approach can be offered to patients who are very concern with a visible surgical scar on their face and neck. Scar is particularly an important consideration for patients who have colored skin type or history of keloid formation. We can now offer small access postaural incision to our patients for their choice.

With wide subcutaneous undermining, the postaural incision alone is adequate to expose the mid and lower pole parotid regions which are the location of most parotid tumors. With undermining of subcutaneous space, the skin flap can be retracted to expose nearly the whole parotid gland (except the most upper and anterior regions), upper half of sternomastoid muscle, whole posterior belly of digastric muscle and facial nerve branches. Wide and adequate undermining of subcutaneous space is the key for exposure. With this keyhole access, the clamp-cut-tie technique is not applicable in controlling hemostasis during dissection of parotid gland, and the radiofrequency bipolar forceps can help in both coagulation and cutting of parotid tissues with adequate hemostasis and safety in avoiding facial nerve damage. Surgical clip is used in transection of parotid vein.

The small access postaural approach, however, has limited exposure to upper pole and far anterior region of the parotid gland. Preaural extension along the pre-lobule sulcus and tip of tragal cartilage may be necessary as decided intraoperatively to gain exposure to upper pole and far anterior areas. For some patients with large size tumor in the lower pole, hairline extension posteriorly can be done as decided intraoperatively to gain exposure to the lower pole. For the more difficult cases, both preauricular and hairline extension can be added simultaneously in converting to a full modified facelift approach to gain a much wider exposure to all regions of the parotid.

There were three main factors affecting the chance of successful postaural small access approach including size of tumor, location of tumor and difficulty of dissection. Tumor size of 6 cm or larger is probably too large for this approach. The postaural approach has adequate exposure for small to medium size benign tumors up to 6 cm in the central and lower superficial lobe of the parotid gland. It would be more difficult to remove tumors located in more anterior part or upper part of parotid; these surgical fields were further away from the postaural wound. Dissection in the deep lobe was technically more demanding and much wider exposure was often necessary. This approach is usually not suitable for malignant tumors in which a much radical surgery with wider resection margin is necessary. Postaural approach can be performed only in exceptional malignant tumors which are small and are located in well-exposed superficial lobe. In the present series, only one small size (2.5 cm) lower pole superficial lobe low grade mucoepidermoid carcinoma was feasible for minimally invasive postaural approach.

In the practice of parotidectomy, 71 % could be done with the small postaural wound. The other 28 % parotidectomy were done as decided pre-operatively or intraoperatively as appropriate using postaural approach with extensions, modified facelift incision, Blair incision and transoral incision. There are also many other approaches and techniques used in my practice of parotidectomy which are not covered in this period of study including maxillary swing approach, modified facelift incision with temporal and/or neck extension, mandibulotomy, mastoidectomy or a combination the them. With more options and flexibility in our surgical armamentarium, we can offer the most appropriate surgical treatment to our patients with due considerations of the disease nature, size, location, safety of the surgery, ethnic background and the high demand of minimal scar and deformity by the patients.

## Conclusion

The small access postaural approach is a further milestone in cosmetic consideration of parotidectomy with advantage of no visible scar on the face and neck. It is a feasible and safe approach for most small to medium size benign parotid tumors in the central and lower pole regions of the parotid gland. It is versatile and can be extended readily to preaural and/or hairline regions to increase exposure, as decided intraoperatively whenever necessary, without compromising the surgical exposure and risk to the patient.
